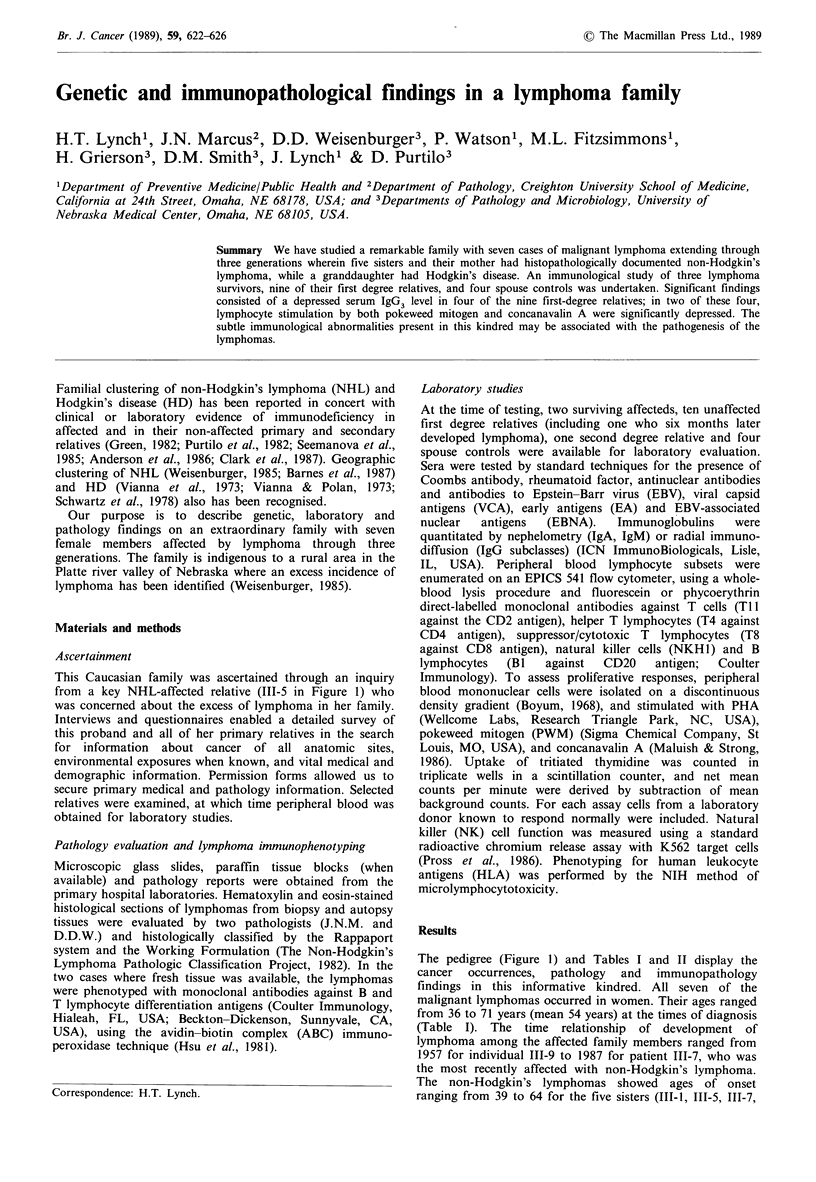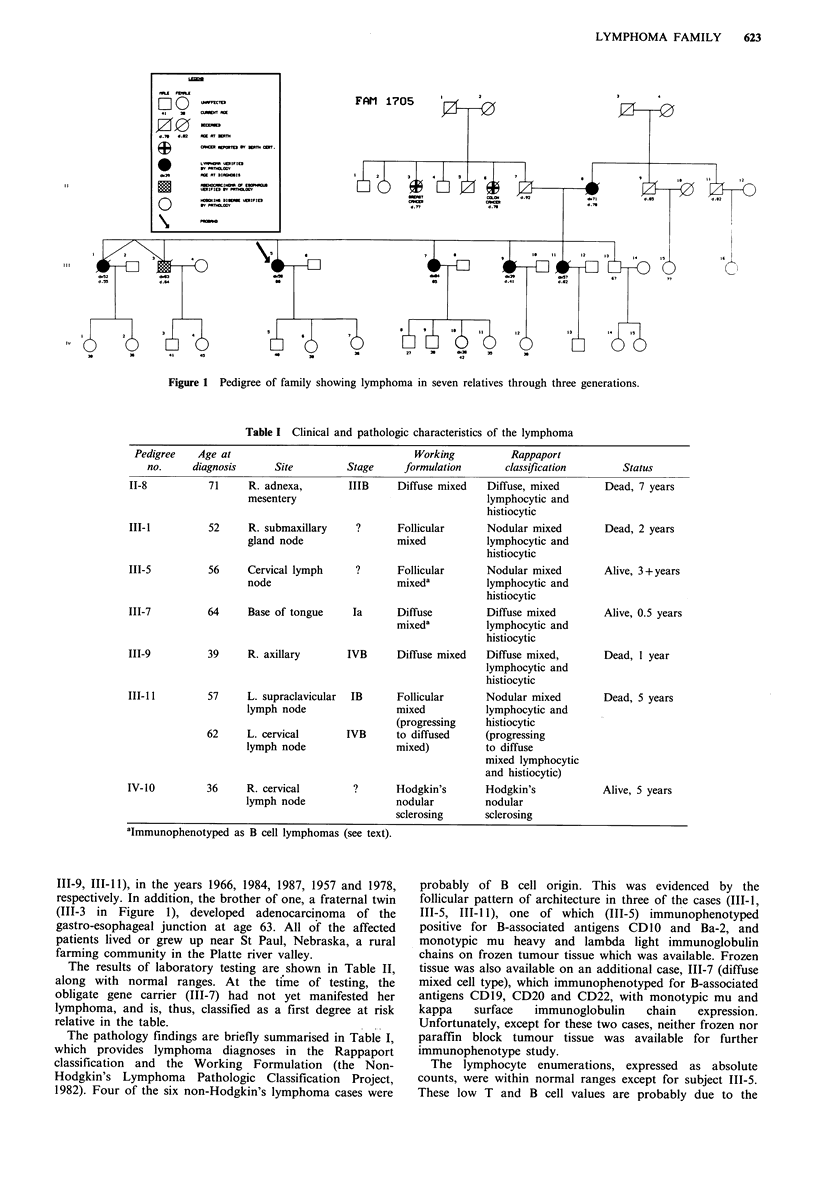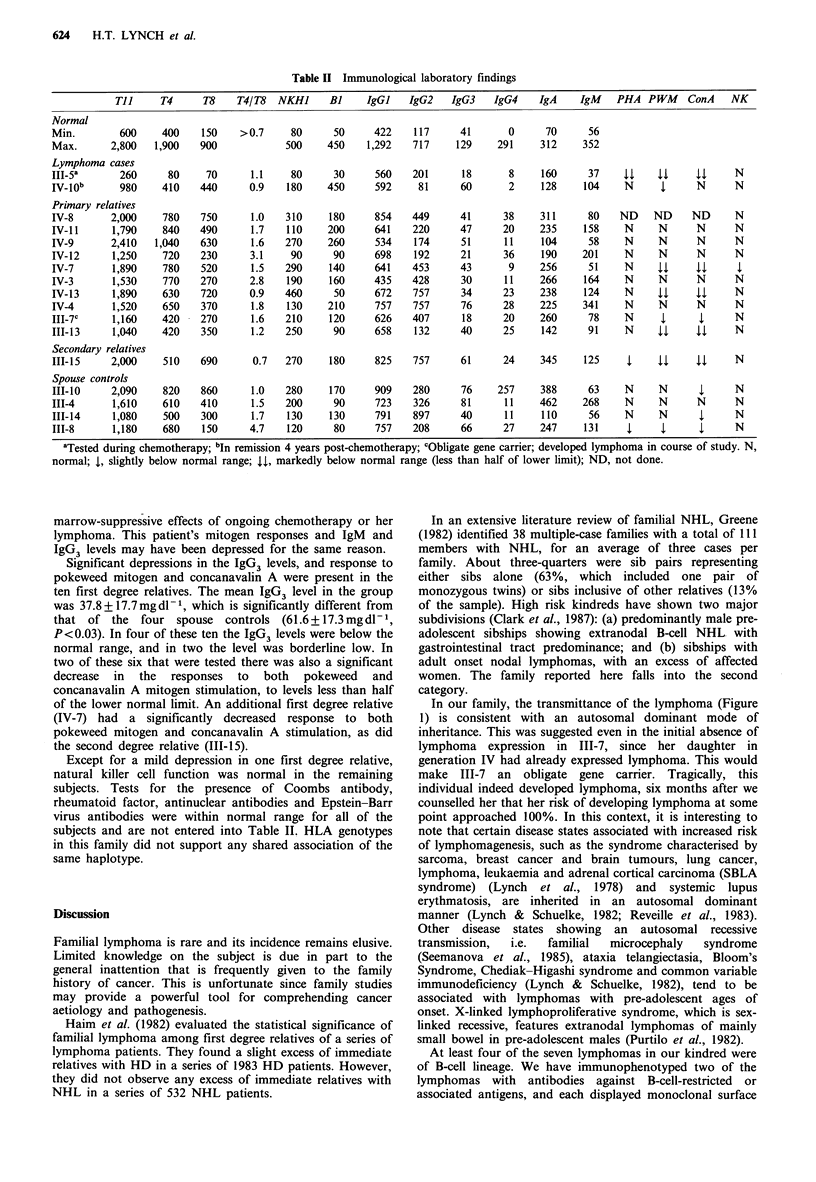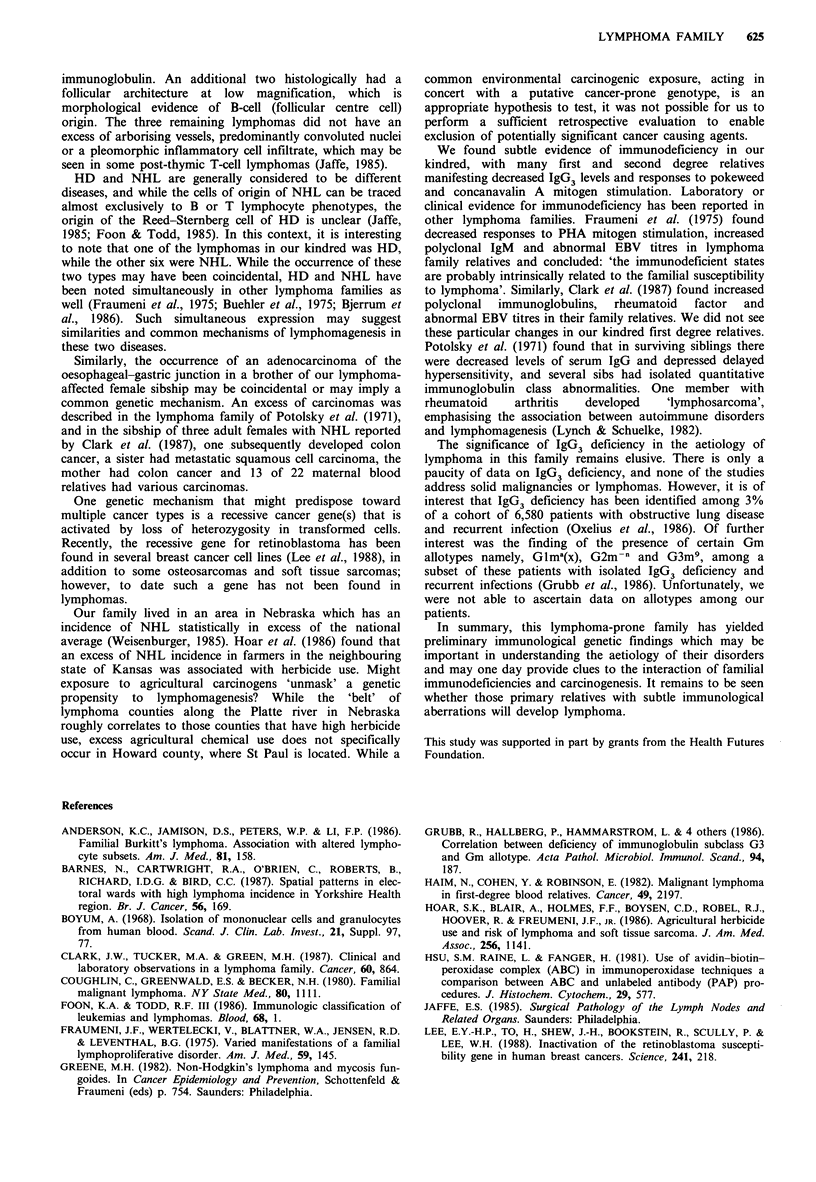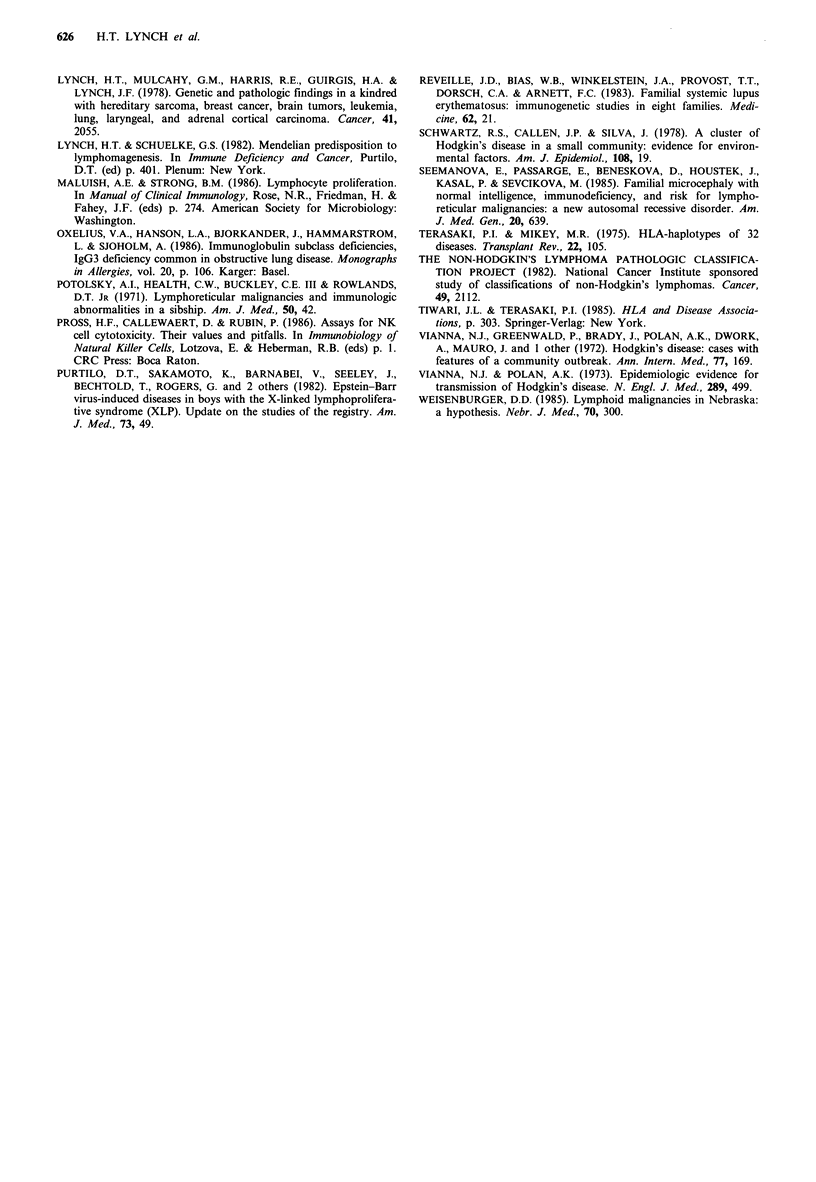# Genetic and immunopathological findings in a lymphoma family.

**DOI:** 10.1038/bjc.1989.126

**Published:** 1989-04

**Authors:** H. T. Lynch, J. N. Marcus, D. D. Weisenburger, P. Watson, M. L. Fitzsimmons, H. Grierson, D. M. Smith, J. Lynch, D. Purtilo

**Affiliations:** Department of Preventive Medicine/Public Health, Creighton University School of Medicine, Omaha, NE 68178.

## Abstract

We have studied a remarkable family with seven cases of malignant lymphoma extending through three generations wherein five sisters and their mother had histopathologically documented non-Hodgkin's lymphoma, while a granddaughter had Hodgkin's disease. An immunological study of three lymphoma survivors, nine of their first degree relatives, and four spouse controls was undertaken. Significant findings consisted of a depressed serum IgG3 level in four of the nine first-degree relatives; in two of these four, lymphocyte stimulation by both pokeweed mitogen and concanavalin A were significantly depressed. The subtle immunological abnormalities present in this kindred may be associated with the pathogenesis of the lymphomas.


*Br. J. Cancer* (1989), **59**, 622–626 © The Macmillan Press Ltd., 1989

### Genetic and immunopathological findings in a lymphoma family

H.T. Lynch[1], J.N. Marcus[2], D.D. Weisenburger[3], P. Watson[1], M.L. Fitzsimmons[1], H. Grierson[3], D.M. Smith[3], J. Lynch[1] & D. Purtilo[3]

[1]*Department of Preventive Medicine/Public Health and* [2]*Department of Pathology, Creighton University School of Medicine, California at 24th Street, Omaha, NE 68178, USA; and* [3]*Departments of Pathology and Microbiology, University of Nebraska Medical Center, Omaha, NE 68105, USA.*

**Summary** We have studied a remarkable family with seven cases of malignant lymphoma extending through three generations wherein five sisters and their mother had histopathologically documented non-Hodgkin's lymphoma, while a granddaughter had Hodgkin's disease. An immunological study of three lymphoma survivors, nine of their first degree relatives, and four spouse controls was undertaken. Significant findings consisted of a depressed serum $IgG_3$ level in four of the nine first-degree relatives; in two of these four, lymphocyte stimulation by both pokeweed mitogen and concanavalin A were significantly depressed. The subtle immunological abnormalities present in this kindred may be associated with the pathogenesis of the lymphomas.

Familial clustering of non-Hodgkin's lymphoma (NHL) and Hodgkin's disease (HD) has been reported in concert with clinical or laboratory evidence of immunodeficiency in affected and in their non-affected primary and secondary relatives (Green, 1982; Purtilo *et al.*, 1982; Seemanova *et al.*, 1985; Anderson *et al.*, 1986; Clark *et al.*, 1987). Geographic clustering of NHL (Weisenburger, 1985; Barnes *et al.*, 1987) and HD (Vianna *et al.*, 1973; Vianna & Polan, 1973; Schwartz *et al.*, 1978) also has been recognised.

Our purpose is to describe genetic, laboratory and pathology findings on an extraordinary family with seven female members affected by lymphoma through three generations. The family is indigenous to a rural area in the Platte river valley of Nebraska where an excess incidence of lymphoma has been identified (Weisenburger, 1985).

### Materials and methods

### *Ascertainment*

This Caucasian family was ascertained through an inquiry from a key NHL-affected relative (III-5 in Figure 1) who was concerned about the excess of lymphoma in her family. Interviews and questionnaires enabled a detailed survey of this proband and all of her primary relatives in the search for information about cancer of all anatomic sites, environmental exposures when known, and vital medical and demographic information. Permission forms allowed us to secure primary medical and pathology information. Selected relatives were examined, at which time peripheral blood was obtained for laboratory studies.

### *Pathology evaluation and lymphoma immunophenotyping*

Microscopic glass slides, paraffin tissue blocks (when available) and pathology reports were obtained from the primary hospital laboratories. Hematoxylin and eosin-stained histological sections of lymphomas from biopsy and autopsy tissues were evaluated by two pathologists (J.N.M. and D.D.W.) and histologically classified by the Rappaport system and the Working Formulation (The Non-Hodgkin's Lymphoma Pathologic Classification Project, 1982). In the two cases where fresh tissue was available, the lymphomas were phenotyped with monoclonal antibodies against B and T lymphocyte differentiation antigens (Coulter Immunology, Hialeah, FL, USA; Beckton–Dickenson, Sunnyvale, CA, USA), using the avidin–biotin complex (ABC) immunoperoxidase technique (Hsu *et al.*, 1981).

Correspondence: H.T. Lynch.

### *Laboratory studies*

At the time of testing, two surviving affecteds, ten unaffected first degree relatives (including one who six months later developed lymphoma), one second degree relative and four spouse controls were available for laboratory evaluation. Sera were tested by standard techniques for the presence of Coombs antibody, rheumatoid factor, antinuclear antibodies and antibodies to Epstein–Barr virus (EBV), viral capsid antigens (VCA), early antigens (EA) and EBV-associated nuclear antigens (EBNA). Immunoglobulins were quantitated by nephelometry (IgA, IgM) or radial immunodiffusion (IgG subclasses) (ICN ImmunoBiologicals, Lisle, IL, USA). Peripheral blood lymphocyte subsets were enumerated on an EPICS 541 flow cytometer, using a whole-blood lysis procedure and fluorescein or phycoerythrin direct-labelled monoclonal antibodies against T cells (T11 against the CD2 antigen), helper T lymphocytes (T4 against CD4 antigen), suppressor/cytotoxic T lymphocytes (T8 against CD8 antigen), natural killer cells (NKH1) and B lymphocytes (B1 against CD20 antigen; Coulter Immunology). To assess proliferative responses, peripheral blood mononuclear cells were isolated on a discontinuous density gradient (Boyum, 1968), and stimulated with PHA (Wellcome Labs, Research Triangle Park, NC, USA), pokeweed mitogen (PWM) (Sigma Chemical Company, St Louis, MO, USA), and concanavalin A (Maluish & Strong, 1986). Uptake of tritiated thymidine was counted in triplicate wells in a scintillation counter, and net mean counts per minute were derived by subtraction of mean background counts. For each assay cells from a laboratory donor known to respond normally were included. Natural killer (NK) cell function was measured using a standard radioactive chromium release assay with K562 target cells (Pross *et al.*, 1986). Phenotyping for human leukocyte antigens (HLA) was performed by the NIH method of microlymphocytotoxicity.

### Results

The pedigree (Figure 1) and Tables I and II display the cancer occurrences, pathology and immunopathology findings in this informative kindred. All seven of the malignant lymphomas occurred in women. Their ages ranged from 36 to 71 years (mean 54 years) at the times of diagnosis (Table I). The time relationship of development of lymphoma among the affected family members ranged from 1957 for individual III-9 to 1987 for patient III-7, who was the most recently affected with non-Hodgkin's lymphoma. The non-Hodgkin's lymphomas showed ages of onset ranging from 39 to 64 for the five sisters (III-1, III-5, III-7,

**Figure 1** Pedigree of family showing lymphoma in seven relatives through three generations.

**Table I** Clinical and pathologic characteristics of the lymphoma

| *Pedigree no.* | *Age at diagnosis* | *Site* | *Stage* | *Working formulation* | *Rappaport classification* | *Status* |
|---|---|---|---|---|---|---|
| II-8 | 71 | R. adnexa, mesentery | IIIB | Diffuse mixed | Diffuse, mixed lymphocytic and histiocytic | Dead, 7 years |
| III-1 | 52 | R. submaxillary gland node | ? | Follicular mixed | Nodular mixed lymphocytic and histiocytic | Dead, 2 years |
| III-5 | 56 | Cervical lymph node | ? | Follicular mixed[a] | Nodular mixed lymphocytic and histiocytic | Alive, 3+years |
| III-7 | 64 | Base of tongue | Ia | Diffuse mixed[a] | Diffuse mixed lymphocytic and histiocytic | Alive, 0.5 years |
| III-9 | 39 | R. axillary | IVB | Diffuse mixed | Diffuse mixed, lymphocytic and histiocytic | Dead, 1 year |
| III-11 | 57 | L. supraclavicular lymph node | IB | Follicular mixed (progressing to diffused mixed) | Nodular mixed lymphocytic and histiocytic (progressing to diffuse mixed lymphocytic and histiocytic) | Dead, 5 years |
| | 62 | L. cervical lymph node | IVB | | | |
| IV-10 | 36 | R. cervical lymph node | ? | Hodgkin's nodular sclerosing | Hodgkin's nodular sclerosing | Alive, 5 years |

[a]Immunophenotyped as B cell lymphomas (see text).

III-9, III-11), in the years 1966, 1984, 1987, 1957 and 1978, respectively. In addition, the brother of one, a fraternal twin (III-3 in Figure 1), developed adenocarcinoma of the gastro-esophageal junction at age 63. All of the affected patients lived or grew up near St Paul, Nebraska, a rural farming community in the Platte river valley.

The results of laboratory testing are shown in Table II, along with normal ranges. At the time of testing, the obligate gene carrier (III-7) had not yet manifested her lymphoma, and is, thus, classified as a first degree at risk relative in the table.

The pathology findings are briefly summarised in Table I, which provides lymphoma diagnoses in the Rappaport classification and the Working Formulation (the Non-Hodgkin's Lymphoma Pathologic Classification Project, 1982). Four of the six non-Hodgkin's lymphoma cases were probably of B cell origin. This was evidenced by the follicular pattern of architecture in three of the cases (III-1, III-5, III-11), one of which (III-5) immunophenotyped positive for B-associated antigens CD10 and Ba-2, and monotypic mu heavy and lambda light immunoglobulin chains on frozen tumour tissue which was available. Frozen tissue was also available on an additional case, III-7 (diffuse mixed cell type), which immunophenotyped for B-associated antigens CD19, CD20 and CD22, with monotypic mu and kappa surface immunoglobulin chain expression. Unfortunately, except for these two cases, neither frozen nor paraffin block tumour tissue was available for further immunophenotype study.

The lymphocyte enumerations, expressed as absolute counts, were within normal ranges except for subject III-5. These low T and B cell values are probably due to the

**Table II** Immunological laboratory findings

| | *T11* | *T4* | *T8* | *T4/T8* | *NKH1* | *B1* | *IgG1* | *IgG2* | *IgG3* | *IgG4* | *IgA* | *IgM* | *PHA* | *PWM* | *ConA* | *NK* |
|---|---|---|---|---|---|---|---|---|---|---|---|---|---|---|---|---|
| *Normal* | | | | | | | | | | | | | | | | |
| Min. | 600 | 400 | 150 | >0.7 | 80 | 50 | 422 | 117 | 41 | 0 | 70 | 56 | | | | |
| Max. | 2,800 | 1,900 | 900 | | 500 | 450 | 1,292 | 717 | 129 | 291 | 312 | 352 | | | | |
| *Lymphoma cases* | | | | | | | | | | | | | | | | |
| III-5[a] | 260 | 80 | 70 | 1.1 | 80 | 30 | 560 | 201 | 18 | 8 | 160 | 37 | ↓↓ | ↓↓ | ↓↓ | N |
| IV-10[b] | 980 | 410 | 440 | 0.9 | 180 | 450 | 592 | 81 | 60 | 2 | 128 | 104 | N | ↓ | N | N |
| *Primary relatives* | | | | | | | | | | | | | | | | |
| IV-8 | 2,000 | 780 | 750 | 1.0 | 310 | 180 | 854 | 449 | 41 | 38 | 311 | 80 | ND | ND | ND | N |
| IV-11 | 1,790 | 840 | 490 | 1.7 | 110 | 200 | 641 | 220 | 47 | 20 | 235 | 158 | N | N | N | N |
| IV-9 | 2,410 | 1,040 | 630 | 1.6 | 270 | 260 | 534 | 174 | 51 | 11 | 104 | 58 | N | N | N | N |
| IV-12 | 1,250 | 720 | 230 | 3.1 | 90 | 90 | 698 | 192 | 21 | 36 | 190 | 201 | N | N | N | N |
| IV-7 | 1,890 | 780 | 520 | 1.5 | 290 | 140 | 641 | 453 | 43 | 9 | 256 | 51 | N | ↓↓ | ↓↓ | ↓ |
| IV-3 | 1,530 | 770 | 270 | 2.8 | 190 | 160 | 435 | 428 | 30 | 11 | 266 | 164 | N | N | N | N |
| IV-13 | 1,890 | 630 | 720 | 0.9 | 460 | 50 | 672 | 757 | 34 | 23 | 238 | 124 | N | ↓↓ | ↓↓ | N |
| IV-4 | 1,520 | 650 | 370 | 1.8 | 130 | 210 | 757 | 757 | 76 | 28 | 225 | 341 | N | N | N | N |
| III-7[c] | 1,160 | 420 | 270 | 1.6 | 210 | 120 | 626 | 407 | 18 | 20 | 260 | 78 | N | ↓ | ↓ | N |
| III-13 | 1,040 | 420 | 350 | 1.2 | 250 | 90 | 658 | 132 | 40 | 25 | 142 | 91 | N | ↓↓ | ↓↓ | N |
| *Secondary relatives* | | | | | | | | | | | | | | | | |
| III-15 | 2,000 | 510 | 690 | 0.7 | 270 | 180 | 825 | 757 | 61 | 24 | 345 | 125 | ↓ | ↓↓ | ↓↓ | N |
| *Spouse controls* | | | | | | | | | | | | | | | | |
| III-10 | 2,090 | 820 | 860 | 1.0 | 280 | 170 | 909 | 280 | 76 | 257 | 388 | 63 | N | N | ↓ | N |
| III-4 | 1,610 | 610 | 410 | 1.5 | 200 | 90 | 723 | 326 | 81 | 11 | 462 | 268 | N | N | N | N |
| III-14 | 1,080 | 500 | 300 | 1.7 | 130 | 130 | 791 | 897 | 40 | 11 | 110 | 56 | N | N | ↓ | N |
| III-8 | 1,180 | 680 | 150 | 4.7 | 120 | 80 | 757 | 208 | 66 | 27 | 247 | 131 | ↓ | ↓ | ↓ | N |

[a]Tested during chemotherapy; [b]In remission 4 years post-chemotherapy; [c]Obligate gene carrier; developed lymphoma in course of study. N, normal; ↓, slightly below normal range; ↓↓, markedly below normal range (less than half of lower limit); ND, not done.

marrow-suppressive effects of ongoing chemotherapy or her lymphoma. This patient's mitogen responses and IgM and $IgG_3$ levels may have been depressed for the same reason.

Significant depressions in the $IgG_3$ levels, and response to pokeweed mitogen and concanavalin A were present in the ten first degree relatives. The mean $IgG_3$ level in the group was $37.8 \pm 17.7 \, mg \, dl^{-1}$, which is significantly different from that of the four spouse controls ($61.6 \pm 17.3 \, mg \, dl^{-1}$, $P < 0.03$). In four of these ten the $IgG_3$ levels were below the normal range, and in two the level was borderline low. In two of these six that were tested there was also a significant decrease in the responses to both pokeweed and concanavalin A mitogen stimulation, to levels less than half of the lower normal limit. An additional first degree relative (IV-7) had a significantly decreased response to both pokeweed mitogen and concanavalin A stimulation, as did the second degree relative (III-15).

Except for a mild depression in one first degree relative, natural killer cell function was normal in the remaining subjects. Tests for the presence of Coombs antibody, rheumatoid factor, antinuclear antibodies and Epstein–Barr virus antibodies were within normal range for all of the subjects and are not entered into Table II. HLA genotypes in this family did not support any shared association of the same haplotype.

### Discussion

Familial lymphoma is rare and its incidence remains elusive. Limited knowledge on the subject is due in part to the general inattention that is frequently given to the family history of cancer. This is unfortunate since family studies may provide a powerful tool for comprehending cancer aetiology and pathogenesis.

Haim *et al.* (1982) evaluated the statistical significance of familial lymphoma among first degree relatives of a series of lymphoma patients. They found a slight excess of immediate relatives with HD in a series of 1983 HD patients. However, they did not observe any excess of immediate relatives with NHL in a series of 532 NHL patients.

In an extensive literature review of familial NHL, Greene (1982) identified 38 multiple-case families with a total of 111 members with NHL, for an average of three cases per family. About three-quarters were sib pairs representing either sibs alone (63%, which included one pair of monozygous twins) or sibs inclusive of other relatives (13% of the sample). High risk kindreds have shown two major subdivisions (Clark *et al.*, 1987): (a) predominantly male pre-adolescent sibships showing extranodal B-cell NHL with gastrointestinal tract predominance; and (b) sibships with adult onset nodal lymphomas, with an excess of affected women. The family reported here falls into the second category.

In our family, the transmittance of the lymphoma (Figure 1) is consistent with an autosomal dominant mode of inheritance. This was suggested even in the initial absence of lymphoma expression in III-7, since her daughter in generation IV had already expressed lymphoma. This would make III-7 an obligate gene carrier. Tragically, this individual indeed developed lymphoma, six months after we counselled her that her risk of developing lymphoma at some point approached 100%. In this context, it is interesting to note that certain disease states associated with increased risk of lymphomagenesis, such as the syndrome characterised by sarcoma, breast cancer and brain tumours, lung cancer, lymphoma, leukaemia and adrenal cortical carcinoma (SBLA syndrome) (Lynch *et al.*, 1978) and systemic lupus erythmatosis, are inherited in an autosomal dominant manner (Lynch & Schuelke, 1982; Reveille *et al.*, 1983). Other disease states showing an autosomal recessive transmission, i.e. familial microcephaly syndrome (Seemanova *et al.*, 1985), ataxia telangiectasia, Bloom's Syndrome, Chediak–Higashi syndrome and common variable immunodeficiency (Lynch & Schuelke, 1982), tend to be associated with lymphomas with pre-adolescent ages of onset. X-linked lymphoproliferative syndrome, which is sex-linked recessive, features extranodal lymphomas of mainly small bowel in pre-adolescent males (Purtilo *et al.*, 1982).

At least four of the seven lymphomas in our kindred were of B-cell lineage. We have immunophenotyped two of the lymphomas with antibodies against B-cell-restricted or associated antigens, and each displayed monoclonal surface immunoglobulin. An additional two histologically had a follicular architecture at low magnification, which is morphological evidence of B-cell (follicular centre cell) origin. The three remaining lymphomas did not have an excess of arborising vessels, predominantly convoluted nuclei or a pleomorphic inflammatory cell infiltrate, which may be seen in some post-thymic T-cell lymphomas (Jaffe, 1985).

HD and NHL are generally considered to be different diseases, and while the cells of origin of NHL can be traced almost exclusively to B or T lymphocyte phenotypes, the origin of the Reed–Sternberg cell of HD is unclear (Jaffe, 1985; Foon & Todd, 1985). In this context, it is interesting to note that one of the lymphomas in our kindred was HD, while the other six were NHL. While the occurrence of these two types may have been coincidental, HD and NHL have been noted simultaneously in other lymphoma families as well (Fraumeni *et al*., 1975; Buehler *et al*., 1975; Bjerrum *et al*., 1986). Such simultaneous expression may suggest similarities and common mechanisms of lymphomagenesis in these two diseases.

Similarly, the occurrence of an adenocarcinoma of the oesophageal–gastric junction in a brother of our lymphoma-affected female sibship may be coincidental or may imply a common genetic mechanism. An excess of carcinomas was described in the lymphoma family of Potolsky *et al*. (1971), and in the sibship of three adult females with NHL reported by Clark *et al*. (1987), one subsequently developed colon cancer, a sister had metastatic squamous cell carcinoma, the mother had colon cancer and 13 of 22 maternal blood relatives had various carcinomas.

One genetic mechanism that might predispose toward multiple cancer types is a recessive cancer gene(s) that is activated by loss of heterozygosity in transformed cells. Recently, the recessive gene for retinoblastoma has been found in several breast cancer cell lines (Lee *et al*., 1988), in addition to some osteosarcomas and soft tissue sarcomas; however, to date such a gene has not been found in lymphomas.

Our family lived in an area in Nebraska which has an incidence of NHL statistically in excess of the national average (Weisenburger, 1985). Hoar *et al*. (1986) found that an excess of NHL incidence in farmers in the neighbouring state of Kansas was associated with herbicide use. Might exposure to agricultural carcinogens 'unmask' a genetic propensity to lymphomagenesis? While the 'belt' of lymphoma counties along the Platte river in Nebraska roughly correlates to those counties that have high herbicide use, excess agricultural chemical use does not specifically occur in Howard county, where St Paul is located. While a common environmental carcinogenic exposure, acting in concert with a putative cancer-prone genotype, is an appropriate hypothesis to test, it was not possible for us to perform a sufficient retrospective evaluation to enable exclusion of potentially significant cancer causing agents.

We found subtle evidence of immunodeficiency in our kindred, with many first and second degree relatives manifesting decreased $IgG_3$ levels and responses to pokeweed and concanavalin A mitogen stimulation. Laboratory or clinical evidence for immunodeficiency has been reported in other lymphoma families. Fraumeni *et al*. (1975) found decreased responses to PHA mitogen stimulation, increased polyclonal IgM and abnormal EBV titres in lymphoma family relatives and concluded: 'the immunodeficient states are probably intrinsically related to the familial susceptibility to lymphoma'. Similarly, Clark *et al*. (1987) found increased polyclonal immunoglobulins, rheumatoid factor and abnormal EBV titres in their family relatives. We did not see these particular changes in our kindred first degree relatives. Potolsky *et al*. (1971) found that in surviving siblings there were decreased levels of serum IgG and depressed delayed hypersensitivity, and several sibs had isolated quantitative immunoglobulin class abnormalities. One member with rheumatoid arthritis developed 'lymphosarcoma', emphasising the association between autoimmune disorders and lymphomagenesis (Lynch & Schuelke, 1982).

The significance of $IgG_3$ deficiency in the aetiology of lymphoma in this family remains elusive. There is only a paucity of data on $IgG_3$ deficiency, and none of the studies address solid malignancies or lymphomas. However, it is of interest that $IgG_3$ deficiency has been identified among 3% of a cohort of 6,580 patients with obstructive lung disease and recurrent infection (Oxelius *et al*., 1986). Of further interest was the finding of the presence of certain Gm allotypes namely, $G1m^{a}(x)$, $G2m^{-n}$ and $G3m^{g}$, among a subset of these patients with isolated $IgG_3$ deficiency and recurrent infections (Grubb *et al*., 1986). Unfortunately, we were not able to ascertain data on allotypes among our patients.

In summary, this lymphoma-prone family has yielded preliminary immunological genetic findings which may be important in understanding the aetiology of their disorders and may one day provide clues to the interaction of familial immunodeficiencies and carcinogenesis. It remains to be seen whether those primary relatives with subtle immunological aberrations will develop lymphoma.

This study was supported in part by grants from the Health Futures Foundation.

### References

ANDERSON, K.C., JAMISON, D.S., PETERS, W.P. & LI, F.P. (1986). Familial Burkitt's lymphoma. Association with altered lymphocyte subsets. *Am. J. Med.*, **81**, 158.

BARNES, N., CARTWRIGHT, R.A., O'BRIEN, C., ROBERTS, B., RICHARD, I.D.G. & BIRD, C.C. (1987). Spatial patterns in electoral wards with high lymphoma incidence in Yorkshire Health region. *Br. J. Cancer*, **56**, 169.

BOYUM, A. (1968). Isolation of mononuclear cells and granulocytes from human blood. *Scand. J. Clin. Lab. Invest.*, **21**, Suppl. 97, 77.

CLARK, J.W., TUCKER, M.A. & GREEN, M.H. (1987). Clinical and laboratory observations in a lymphoma family. *Cancer*, **60**, 864.

COUGHLIN, C., GREENWALD, E.S. & BECKER, N.H. (1980). Familial malignant lymphoma. *NY State Med.*, **80**, 1111.

FOON, K.A. & TODD, R.F. III (1986). Immunologic classification of leukemias and lymphomas. *Blood*, **68**, 1.

FRAUMENI, J.F., WERTELECKI, V., BLATTNER, W.A., JENSEN, R.D. & LEVENTHAL, B.G. (1975). Varied manifestations of a familial lymphoproliferative disorder. *Am. J. Med.*, **59**, 145.

GREENE, M.H. (1982). Non-Hodgkin's lymphoma and mycosis fungoides. In *Cancer Epidemiology and Prevention*, Schottenfeld & Fraumeni (eds) p. 754. Saunders: Philadelphia.

GRUBB, R., HALLBERG, P., HAMMARSTROM, L. & 4 others (1986). Correlation between deficiency of immunoglobulin subclass G3 and Gm allotype. *Acta Pathol. Microbiol. Immunol. Scand.*, **94**, 187.

HAIM, N., COHEN, Y. & ROBINSON, E. (1982). Malignant lymphoma in first-degree blood relatives. *Cancer*, **49**, 2197.

HOAR, S.K., BLAIR, A., HOLMES, F.F., BOYSEN, C.D., ROBEL, R.J., HOOVER, R. & FREUMENI, J.F., JR. (1986). Agricultural herbicide use and risk of lymphoma and soft tissue sarcoma. *J. Am. Med. Assoc.*, **256**, 1141.

HSU, S.M. RAINE, L. & FANGER, H. (1981). Use of avidin–biotin–peroxidase complex (ABC) in immunoperoxidase techniques a comparison between ABC and unlabeled antibody (PAP) procedures. *J. Histochem. Cytochem.*, **29**, 577.

JAFFE, E.S. (1985). *Surgical Pathology of the Lymph Nodes and Related Organs*. Saunders: Philadelphia.

LEE, E.Y.-H.P., TO, H., SHEW, J.-H., BOOKSTEIN, R., SCULLY, P. & LEE, W.H. (1988). Inactivation of the retinoblastoma susceptibility gene in human breast cancers. *Science*, **241**, 218.

LYNCH, H.T., MULCAHY, G.M., HARRIS, R.E., GUIRGIS, H.A. & LYNCH, J.F. (1978). Genetic and pathologic findings in a kindred with hereditary sarcoma, breast cancer, brain tumors, leukemia, lung, laryngeal, and adrenal cortical carcinoma. *Cancer*, **41**, 2055.

LYNCH, H.T. & SCHUELKE, G.S. (1982). Mendelian predisposition to lymphomagenesis. In *Immune Deficiency and Cancer*, Purtilo, D.T. (ed) p. 401. Plenum: New York.

MALUISH, A.E. & STRONG, B.M. (1986). Lymphocyte proliferation. In *Manual of Clinical Immunology*, Rose, N.R., Friedman, H. & Fahey, J.F. (eds) p. 274. American Society for Microbiology: Washington.

OXELIUS, V.A., HANSON, L.A., BJORKANDER, J., HAMMARSTROM, L. & SJOHOLM, A. (1986). Immunoglobulin subclass deficiencies, IgG3 deficiency common in obstructive lung disease. *Monographs in Allergies*, vol. 20, p. 106. Karger: Basel.

POTOLSKY, A.I., HEALTH, C.W., BUCKLEY, C.E. III & ROWLANDS, D.T. JR (1971). Lymphoreticular malignancies and immunologic abnormalities in a sibship. *Am. J. Med.*, **50**, 42.

PROSS, H.F., CALLEWAERT, D. & RUBIN, P. (1986). Assays for NK cell cytotoxicity. Their values and pitfalls. In *Immunobiology of Natural Killer Cells*, Lotzova, E. & Heberman, R.B. (eds) p. 1. CRC Press: Boca Raton.

PURTILO, D.T., SAKAMOTO, K., BARNABEI, V., SEELEY, J., BECHTOLD, T., ROGERS, G. and 2 others (1982). Epstein–Barr virus-induced diseases in boys with the X-linked lymphoproliferative syndrome (XLP). Update on the studies of the registry. *Am. J. Med.*, **73**, 49.

REVEILLE, J.D., BIAS, W.B., WINKELSTEIN, J.A., PROVOST, T.T., DORSCH, C.A. & ARNETT, F.C. (1983). Familial systemic lupus erythematosus: immunogenetic studies in eight families. *Medicine*, **62**, 21.

SCHWARTZ, R.S., CALLEN, J.P. & SILVA, J. (1978). A cluster of Hodgkin's disease in a small community: evidence for environmental factors. *Am. J. Epidemiol.*, **108**, 19.

SEEMANOVA, E., PASSARGE, E., BENESKOVA, D., HOUSTEK, J., KASAL, P. & SEVCIKOVA, M. (1985). Familial microcephaly with normal intelligence, immunodeficiency, and risk for lymphoreticular malignancies: a new autosomal recessive disorder. *Am. J. Med. Gen.*, **20**, 639.

TERASAKI, P.I. & MIKEY, M.R. (1975). HLA-haplotypes of 32 diseases. *Transplant Rev.*, **22**, 105.

THE NON-HODGKIN'S LYMPHOMA PATHOLOGIC CLASSIFICATION PROJECT (1982). National Cancer Institute sponsored study of classifications of non-Hodgkin's lymphomas. *Cancer*, **49**, 2112.

TIWARI, J.L. & TERASAKI, P.I. (1985). *HLA and Disease Associations*, p. 303. Springer-Verlag: New York.

VIANNA, N.J., GREENWALD, P., BRADY, J., POLAN, A.K., DWORK, A., MAURO, J. and 1 other (1972). Hodgkin's disease: cases with features of a community outbreak. *Ann. Intern. Med.*, **77**, 169.

VIANNA, N.J. & POLAN, A.K. (1973). Epidemiologic evidence for transmission of Hodgkin's disease. *N. Engl. J. Med.*, **289**, 499.

WEISENBURGER, D.D. (1985). Lymphoid malignancies in Nebraska: a hypothesis. *Nebr. J. Med.*, **70**, 300.